# Editorial: Animal welfare: innovations to increase health and reduce antimicrobial use

**DOI:** 10.3389/fvets.2026.1969348

**Published:** 2026-09-14

**Authors:** Hans A. M. Spoolder, Xavier Manteca, Daniela Battaglia, Jun Bao, Francisco Galindo Maldonado

**Affiliations:** 1 Wageningen Livestock Research, Wageningen University and Research, Wageningen, Netherlands; 2 Facultat de Veterinaria, Universitat Autonoma de Barcelona, Bellaterra, Spain; 3 Food and Agriculture Organization of the United Nations, Rome, Italy; 4 Northeast Agricultural University, Harbin, China; 5 Universidad Nacional Autonoma de Mexico, Mexico City, Mexico

**Keywords:** animal health, animal welfare, antimicrobial use, innovations, livestock production

Animal welfare has become a fundamental aspect of sustainable animal production globally. The importance of animal welfare stems largely from the fact that animals are sentient beings, that is, able to experience positive and negative emotions. Additionally, there is growing evidence that animal health and welfare are closely interconnected. This is particularly relevant as livestock production worldwide faces significant and persistent challenges caused by infectious diseases such as Avian Influenza, African Swine Fever, and Bluetongue, which continue to cause widespread animal suffering, severe economic losses, and public health concerns.

Traditionally, responses to these challenges have relied on biosecurity measures, vaccination strategies, and antimicrobial treatments. While these remain essential, there is widespread recognition that addressing welfare problems that are related to nutrition, environmental conditions and behavior is equally critical in preventing and managing disease. Better welfare practices, particularly those aimed at reducing stress, can enhance animals' immune function, making them less susceptible to infections, especially in cases of less virulent pathogens or endemic diseases. Therefore, this Research Topic explores recent developments linking animal welfare and animal health from a “One Health—One Welfare” perspective with the overall objective of improving livestock farming worldwide.

The Research Topic includes six papers which cover four themes: the consequences of zinc oxide withdrawal for pig welfare (paper one); the potential of probiotics to improve health and welfare (papers two and three); the use of automatic systems to monitor health and welfare (paper four) and the benefits of environmental enrichment for health and welfare (papers five and six).

The authors of the first paper discuss producers' perceptions on the impact of zinc oxide withdrawal from the diet of pigs (Ekiri et al.). Zinc oxide reduces post-weaning diarrhea (PWD) and enhances growth performance, but its use has negative consequences regarding antimicrobial resistance (AMR) and environmental contamination. Its use as feed supplement was prohibited in the European Union from June 2022, and in the United Kingdom from June 2024. UK producers are currently looking for alternatives.

In the second paper, probiotics are explored as a potential antioxidant that may reduce stress and promote growth in monogastric animals (Sumanu et al.). The authors conclude that probiotics may offer a promising nutritional strategy, but that future research should focus on clarifying the mechanisms underlying these effects as well as optimizing dosing strategies. Exploring synergistic combinations with prebiotics and phytogenic additives may also be useful.

The third paper addresses a particular application of probiotics. While many studies have been conducted within an experimental setting, there is a lack of studies under real-farm conditions, especially using a multi-farm approach (Nöllenburg et al.). The goal of this study was to test the effect of a fermented herbal extract (FHE) on welfare and health parameters in rearing and finishing pigs at a commercial farm level. Their findings indicate that the FHE's probiotic may have potential to enhance aspects of welfare, namely reduced prevalence of rearing pigs with tail tissue loss and lower incidence of coughing in fatteners. While these findings are promising, they warrant further investigation and confirmation.

Paper four considers that the development of automatic cough detection system may be useful (Cai et al.). These are well-established for pigs, and in this paper novel technologies for the assessment of chicken cough were further developed and tested. The authors conclude that it provides a 24 h real-time monitoring and multi-level early warning capabilities, offering effective technical support for the early detection and intelligent control of respiratory diseases in poultry.

The fifth paper draws attention to the use of fermentation bed farming systems for the improvement of geese health and welfare (Li et al.). The authors hypothesize that environmental enrichment offered through such a system improves the welfare of the animals and reduces stress responses by promoting natural behavioral expressions. It may also reduce the occurrence of abnormal and destructive behaviors and enhance immune function. They conclude that floor farming, by rearing geese on a fermentation bed, enhances their resistance to transport stress by boosting antioxidant capacity, strengthening immune function, and reducing levels of inflammatory cytokines and stress hormones.

In the sixth and final paper, the effects of an enriched environment on stereotypic behavior is discussed at a neural level (Zhang et al.). The authors conclude that environmental complexity in early life is important for reducing stereotypies in later life. They also consider that pharmacological interventions may reduce this abnormal behavior and recommend that future welfare assessment frameworks may benefit from incorporating neurobiological biomarkers to complement purely behavioral observations.

Taken together, the six papers provide further evidence for the close link between welfare and health, and provide examples of research that addresses the benefits of improving animal welfare so that the resilience of farm animals to everyday challenges increases. At the same time, this Research Topic also highlights the need for further research on the interplay between animal welfare and antimicrobial use. In particular, field studies that aim to quantify the effect of improving animal welfare on the use of antimicrobials are urgently needed.

